# Development and Validation of a Five-RNA–Based Signature and Identification of Candidate Drugs for Neuroblastoma

**DOI:** 10.3389/fgene.2021.685646

**Published:** 2021-10-20

**Authors:** PeiPei Zhang, KeXin Ma, XiaoFei Ke, Liu Liu, Ying Li, YaJuan Liu, YouJun Wang

**Affiliations:** Department of Pediatrics, The Fifth Affiliated Hospital of Zhengzhou University, Zhengzhou University, Zhengzhou, China

**Keywords:** Neuroblastoma, age, Signature, prognosis, drugs

## Abstract

Neuroblastoma (NBL) originating from the sympathetic nervous system is the most prevalent solid tumor in infancy. Although there is sufficient variability in prognosis among different age pyramids, age-related gene expression profiles and biomarkers remain poorly explored. The present study aimed to construct a signature based on differentially expressed genes (DEGs) between two age groups in NBL. Univariate Cox regression, multivariate Cox regression, and LASSO analyses were used to identify the optimal prognostic factors. The prediction ability of the model was assessed using the receiver operating characteristic (ROC) curve and C-index. Functional enrichment analysis was performed using the Kyoto Encyclopedia of Genes and Genomes and gene ontology databases. A total of 1,160 DEGs were identified between the two groups, and 204 DEGs impacted the survival of NBL. Functional enrichment analysis revealed that the DEGs were involved in retinol metabolism, cholesterol metabolism, and glycolysis/gluconeogenesis pathways. Five RNAs, namely F8A3, PDF, ANKRD24, FAXDC2, and TMEM160 were recruited into the signature. They were correlated with COG risk classification, INSS stage, and histology. MYCN amplification was linked to FAXDC2, TMEM160, PDF, and F8A3. The expression levels of ANKRD24, PDF, and TMEM160 were lower in the hyperdiploid groups. Only FAXDC2 levels were different in the different MKI grades. The ROC curve showed that the five-RNA–based signatures effectively predicted the OS of NBL (3-years AUC = 0.791, 5-years AUC = 0.816) in the TARGET cohort. The predictive capability was also validated by the GSE49711 cohort (3-years AUC = 0.851, 5-years AUC = 0.848). The C-index in the TARGET and GSE49711 cohorts was 0.749 and 0.809, respectively. The potential mechanisms of the five RNAs were also explored *via* gene set enrichment analysis, and candidate drugs targeting the five genes, including dabrafenib, vemurafenib, and bafetinib, were screened. In conclusion, we constructed a five-RNA–based signature to predict the survival of NBL and screened candidate agents against NBL.

## Introduction

Neuroblastoma (NBL), a malignant embryonal tumor, is the most prevalent solid tumor of infancy and accounts for approximately 15% of childhood cancer deaths (Davidoff, 2012; Gatta et al., 2014). The clinical presentation and progression of NBL are heterogeneous. Clinical manifestations of NBL often depend on the anatomic location and the predilection site in the abdomen (approximately 65%), which manifests as abdominal distention and constipation (Swift et al., 2018). The outcomes of NBL are diverse from spontaneous regression to relentless progression or from resistance to chemoradiotherapy, stem cell transplantation, and immunotherapy (Maris et al., 2007; Brodeur and Bagatell, 2014; Illhardt et al., 2018). Furthermore, despite complex and intensive treatments, the outcome of NBL remains poor, with a 5-years survival rate of less than 50% (Pinto et al., 2015). Therefore, identifying actionable biomarkers in NBL to assist early diagnosis, risk classification, and treatment is essential.

Age is one of the most prominent clinical prognostic indicators of NBL. A study with more than 110.000 NBL patients showed that patients aged >18 months at diagnosis had a low survival rate (Moroz et al., 2011). A similar result was reported by Schmidt et al., in 2005 (Schmidt et al., 2005). Moreover, age is a critical factor in the Shimada classification system (Shimada et al., 2001). Despite the essential role of age in NBL the underlying biology remains unclear. Accumulated evidence has demonstrated that many abnormalities at the transcriptome level, including DLK, BIRC5, CDKN2D, and SMARCD3 play a role in tumorigenesis, progression, migration, and relapse of NBL (Coco et al., 2009; Tsubota and Kadomatsu, 2018). Analysis of gene expression patterns between age groups has enabled a better understanding of the biology of NBL. According to a previous study, biomarkers identified from gene expression profiles exhibited more effective prediction capability than individual clinical factors (He et al., 2020). Therefore, it is necessary to develop precise computational models based on age-related genes in NBL.

This study analyzed the differentially expressed genes (DEGs) between two age groups and their associated pathways. In addition, we attempted to construct an RNA-based signature to predict the survival of NBL patients in the TARGET cohort using the DEGs.

## Methods and Materials

### Data Source

We downloaded the gene expression profiles and the corresponding clinical information of 153 NBL patients from the TARGET database (www.ocg.cancer.gov/programs/target). The inclusion criteria were based on the data of complete survival rate of patients and the age patients. Another cohort of 489 NBL patients (GSE49711) was obtained from the GEO database (www.ncbi.nlm.nih.gov/geo/) and was used for external validation. Genomics and drug sensitivity data were extracted from the CellMinerCDB database (www.discover.nci.nih.gov/cellminer/home.do).

### Analysis of DEGs

Patients were divided into two subgroups (<18 months and >18 months), and the DEGs between the two groups were analyzed. DEGs with |log2 fold change (FC)| > 1 and false discovery rate (FDR) < 0.05 were identified by the R package “edgR”. These DEGs were then visualized in the volcano plot; the top 30 upregulated and downregulated genes are shown in the heatmap.

### Risk Score Calculation

The univariate Cox regression analysis followed by LASSO regression analysis was done to screen and optimize the prognostic RNAs. Multivariate Cox regression analysis was conducted to calculate the regression coefficients for each gene. The risk score of each sample was calculated according to the following formula: risk score = β1 *X1+ β2 * X2+ … +βnXn (β, regression coefficient; X, prognostic factors). Patients were classified into high-risk and low-risk groups according to the threshold value of the median risk score.

### Kyoto Encyclopedia of Genes and Genomes and Gene Ontology Enrichment Analyses

DEGs between the two age groups were subjected to Kyoto Encyclopedia of Genes and Genomes (KEGG) pathway analysis and the Gene Ontology (GO) function analysis. Both p- and p-adjusted values (<0.05) were deemed to be statistically significant.

### Gene Set Enrichment Analysis

Gene Set Enrichment Analysis (GSEA) was performed using the GSEA software version 4.1.0. Gene set collections including “c2.cp.kegg.v7.2.symbols.gmt” and “c5.go.bp.v7.2.symbols.gmt” were selected to identify the biological terms of the five genes. Terms with |NES| >1, NOM *p*-value < 0.05, and FDR <0.25 were considered significant.

### Statistical Analysis

The Kruskal-Wallis rank-sum test or Wilcoxon rank-sum test measured the relationship between clinical characteristics and RNAs. Prognostic analysis was conducted using univariate and multivariate Cox regression analysess. The Kaplan-Meier (K-M) survival analysis was performed to investigate the correlations between prognostic factors and OS, and differences between groups were tested using the log-rank test. We constructed receiver operating characteristic (ROC) curves and determined the areas under the ROC curve (AUCs). Model discrimination was weighed using the AUCs and C-index. The correlations between genes and drug sensitivity were measured using the Pearson correlation analysis. Data were tested at 5% level of significance (*p* < 0.05). R software (version 3.6.3) with the following packages: “glmnet,” “limma,” “edgeR,” “ggplot2,” and “survivalROC.” was used to analyze the data.

## Results

### Identification of DEGs

**FIGURE 1:**
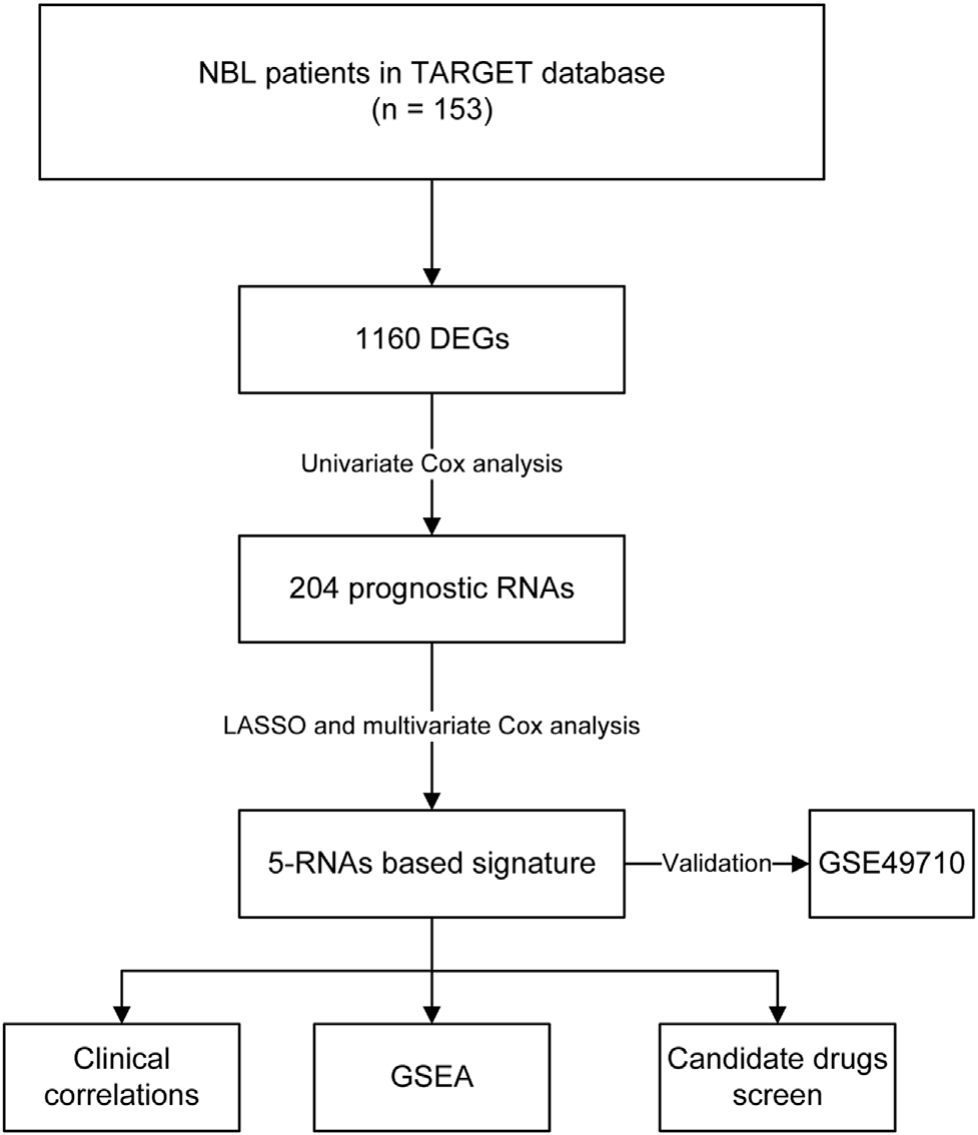
The flow chart of constructing a five-RNA based signature.

**FIGURE 2:**
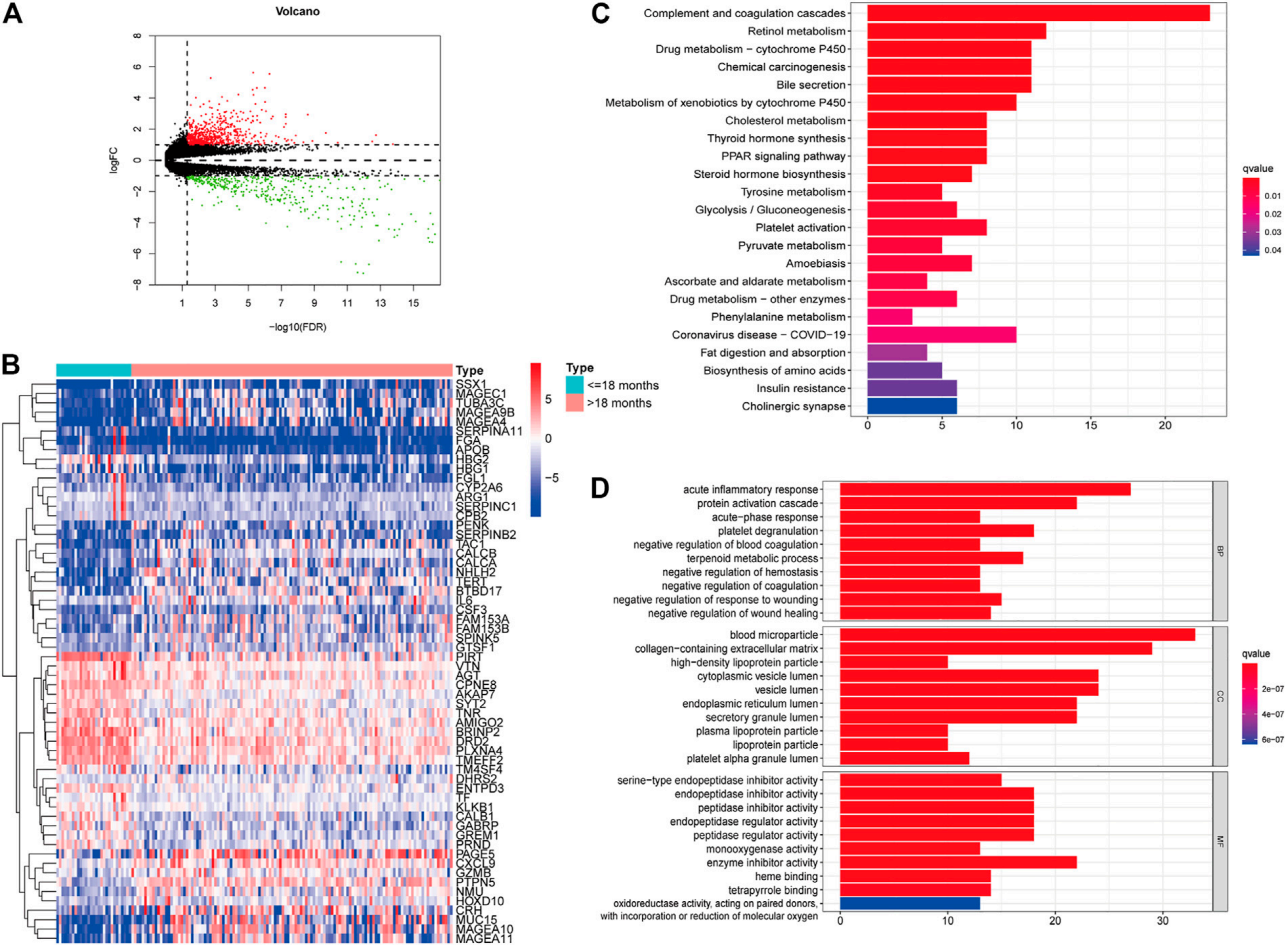
Identification of differently expressed genes (DEGs) in different age groups. Volcano plot of the DEGs in the TARGET **(A)**. Heatmap of the DEGs in the TARGET **(B)**. GO **(C)** and KEGG enrichment analysis **(D)** for 204 DEGs in the TARGET cohort, terms with p- and q-value < 0.05 were considered significant.

We downloaded the transcriptome profiles and corresponding clinical data of 153 NBL patients from the TARGET database. A total of 498 NBL patients from the GSE49711 dataset were used for external validation. The flow chart of the study design is shown in Figure 1. A total of153 NBL patients from the TARGET cohort were divided into two groups of <18 months (n = 29) and >18 months-old NBL patients (n = 124). A total of 1,160 DEGs (678 upregulated and 482 downregulated genes) with |log FC| >1 and FDR <0.05 were identified in the TARGET cohort. These DEGs are shown in the volcano plot, and the most upregulated and downregulated genes are in the heatmap (Figures 2A,B).

Furthermore, univariate Cox regression analysis revealed that 204 out of 1,160 DEGs impacted the survival of NBL patients in the TARGET cohort. KEGG and GO analyses were performed to demonstrate the potential mechanisms of the 204 DEGs. Figure 2C shows that the genes were enhanced in various terms, including complement and coagulation cascades, chemical carcinogenesis, retinol metabolism, and drug metabolism-cytochrome P450.

### Screening Genes for Signature Construction

**FIGURE 3:**
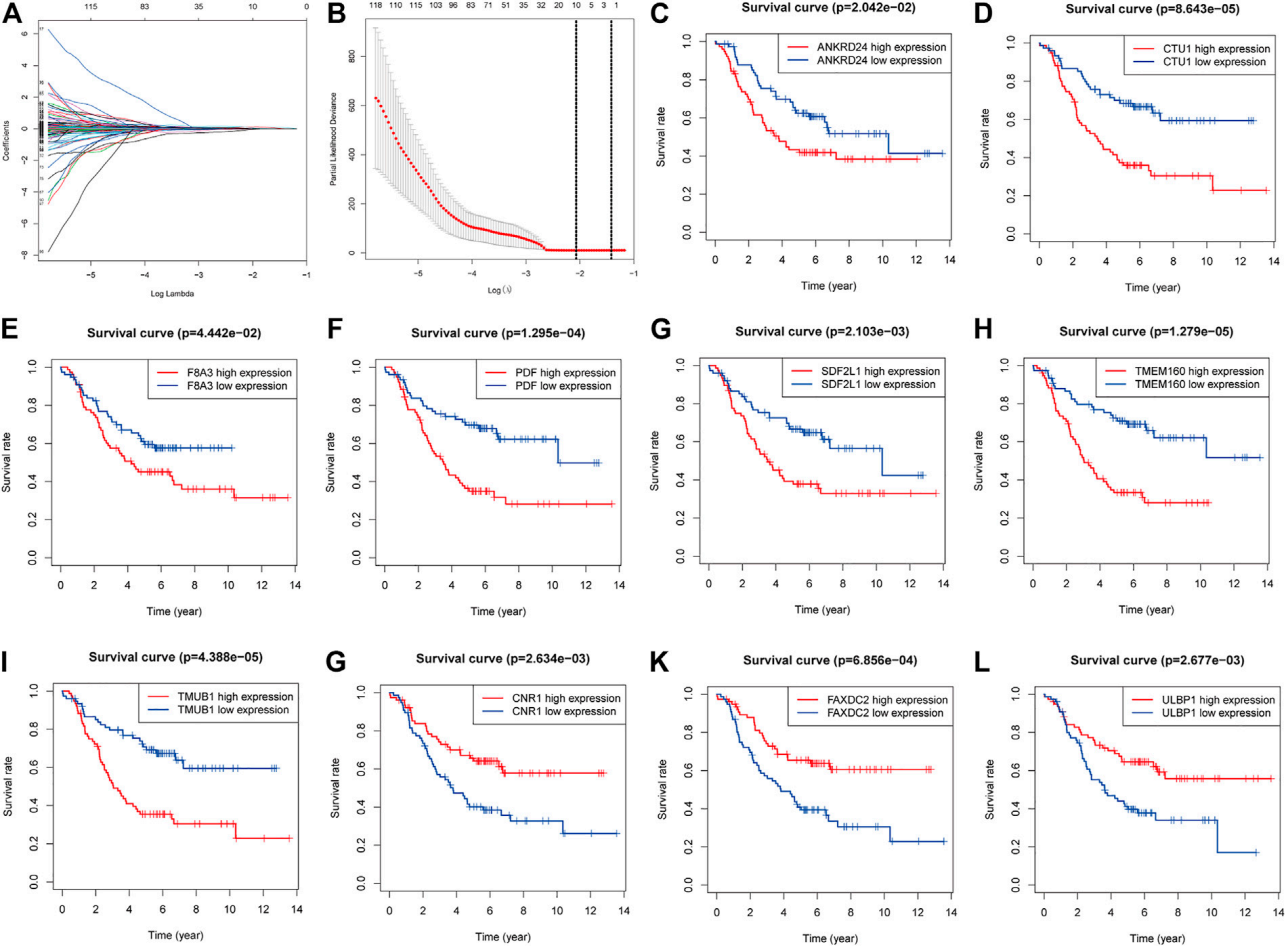
Screening genes to constructing model of predicting the overall survival of NBL and Kaplan–Meier (K-M) survival analysis. **(A)**. Shrinking path diagram of gene entrances into the model. **(B)**. λ selection in the LASSO model used 10-fold cross-validation and λ = 0.132 were selected. The K-M analysis of ANKRD24 **(C)**, CTU1 **(D)**, F8A3 **(E)**, PDF **(F)**, SDF2L1 **(G)**, TMEM160 **(H)**, TMUB1 **(I)**, CNR1 **(G)**, FAXDC2 **(K)**, and ULBP1 **(L)**.

In total, 204 genes were compressed *via* LASSO regression analysis. Figures 3C–I show that when using 10-fold cross-validation, the optimal model was obtained when the lambda was 10, involving 10 genes (TMUB1, CNR1, TMEM160, FAXDC2, SDF2L1, CTU1, PDF, ULBP1, F8A3, and ANKRD24) (Figures 3A,B). K-M survival analysis showed that patients with higher levels of ANKRD24, CTU1, F8A3, PDF, SDF2L1, TMEM160, and TMUB1 (Figures 3C–I) had a lower survival rate compared to those with lower levels. Conversely, patients with elevated expression of CNR1, FAXDC2, and ULBP1 exhibited prolonged survival (Figures 3G–L).

### Correlations Between Ten RNAs and Clinicopathologic Characteristics

**FIGURE 4:**
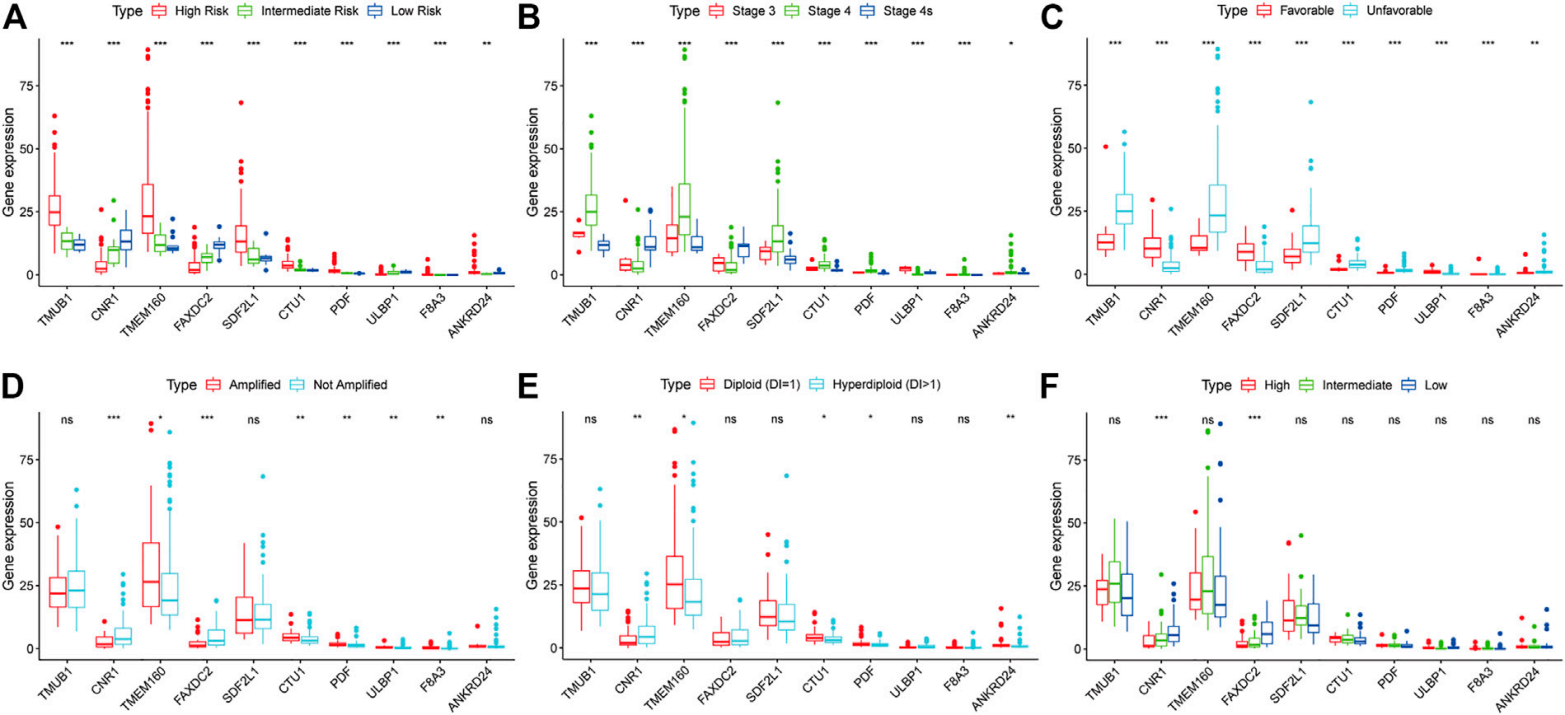
The relationship between the ten RNAs and clinical characteristics. The correlations of the expression of ten genes with the COG classification **(A)**, INSS stage **(B)**, histology group **(C)**, MYCN amplification **(D)**, ploidy status **(E)**, and MKI **(F)**. *p < 0.05, **p < 0.01, ***p < 0.001.

We investigated the relationship between clinical characteristics and the screened 10 RNAs in the TARGET cohort (Supplementary Tables S1–6). Kruskal-Wallis rank-sum test or Wilcoxon rank-sum test showed that the 10 RNAs were differentially expressed in different COG risk classifications, INSS stages, and histology groups (*p* < 0.05; Figures 4A–C). The levels of CNR1 (*p* = 0.001) and FAXDC2 (*p* < 0.001) were lower in MYCN amplification status, whereas the levels of ULBP1 (*p* = 0.002), TMEM160 (*p* = 0.029), CTU1 (*p* = 0.005), PDF (*p* = 0.002), and F8A3 (*p* = 0.007) were higher (Figure 6D). The expression of ANKRD24 (*p* = 0.001), CTU1 (*p* = 0.015), PDF (*p* = 0.032), and TMEM160 (*p* = 0.010) was lower in the hyperdiploid groups except for CNR1 (*p* = 0.004) (Figure 4E). CNR1 (*p* < 0.001) and FAXDC2 (*p* < 0.001) displayed different levels in different MKI grades (Figure 4F).

### Development and Validation of the Five-RNA-Based Signature

**FIGURE 5:**
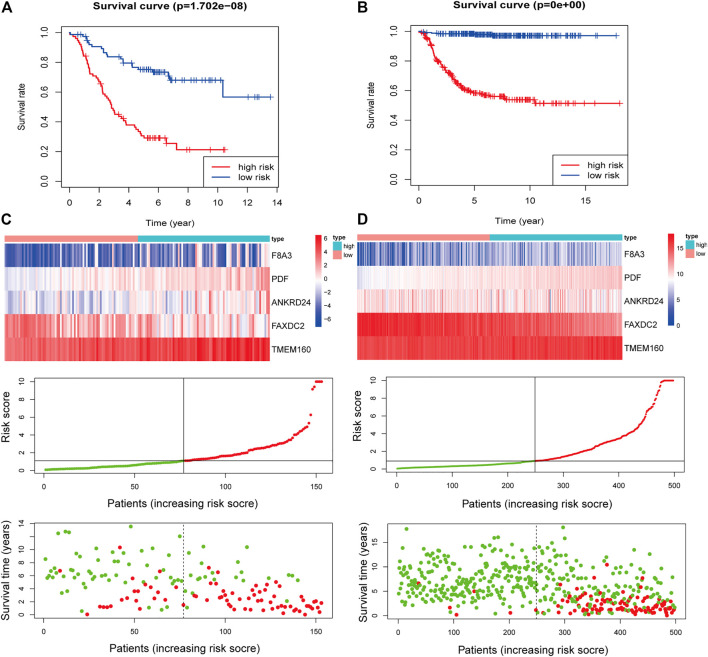
Construction of the five-RNA-based risk signature of neuroblastoma patients. Kaplan-Meier analysis for overall survival (OS) of neuroblastoma patients based on the risk stratification in the TARGET cohort **(A)** and GSE49711 cohort **(B)**. **(C)** The five-RNA-based risk score distribution, the living status of neuroblastoma patients, and heatmap of the five gene expression profiles in the TARGET cohort. **(D)** The five-RNA-based risk score distribution, the living status of neuroblastoma patients, and heatmap of the five gene expression profiles in the GSE49711 cohort.

Multivariate Cox regression analysis showed that F8A3 [HR: 0.808 (95% CI: 0.744–0.872), *p* = 0.001], PDF [HR: 1.879 (95% CI: 1.647–2.110), *p* = 0.007], ANKRD24 [HR: 1.311 (95% CI: 1.209–1.413), *p* = 0.008], FAXDC2 [HR: 0.822 (95% CI: 0.726–0.919), *p* = 0.042], and TMEM160 [HR: 1.507 (95% CI: 1.302–1.712), *p* = 0.046] were independent prognostic factors of NBL (Table 1). Five RNAs (F8A3, PDF, ANKRD24, FAXDC2, and TMEM160) (*p* < 0.05) were included to construct the prediction model based on the Multivariate Cox regression results. The risk score of each patient was calculated according to the formula: Risk score = (0.628 * the expression of TMEM160) + (0.292 * the expression of ANKRD24) + (0.713 * the expression of PDF) + (−0.200 * the expression of F8A3) + (−0.253 * the expression of FAXDC2). Patients were divided into high-risk and low-risk groups based on the median risk score. K-M analysis showed that patients in the low-risk group had significantly longer survival than those in the high-risk group (*p* < 0.001) (Figures 5A,B). The heatmap demonstrated that TMEM160, F8A3, PDF, and ANKRD24 exhibited the highest expression in the high-risk group, whereas FAXDC2 had low expression. Consecutively, mortality was higher in the high-risk group than in the low-risk group (Figure 5C). Similar results were obtained in the GSE49711 cohort, parallel to the TARGET cohort (Figure 5D).

**TABLE 1:** Multivariate Cox regression analysis of ten genes.

Gene	Coef	HR	95% CI	*p*
CNR1	−0.138	0.871	0.791–0.951	0.084
TMEM160	0.410	1.507	1.302–1.712	0.046
TMUB1	0.422	1.525	1.220–1.830	0.167
SDF2L1	0.120	1.127	0.949–1.306	0.502
ANKRD24	0.271	1.311	1.209–1.413	0.008
CTU1	0.175	1.191	0.934–1.447	0.496
ULBP1	−0.018	0.982	0.965–0.999	0.287
PDF	0.631	1.879	1.647–2.110	0.007
F8A3	−0.213	0.808	0.744–0.872	0.001
FAXDC2	−0.196	0.822	0.726–0.919	0.042

HR, Hazard ratio; 95% CI, 95% Confidence interval.

**FIGURE 6:**
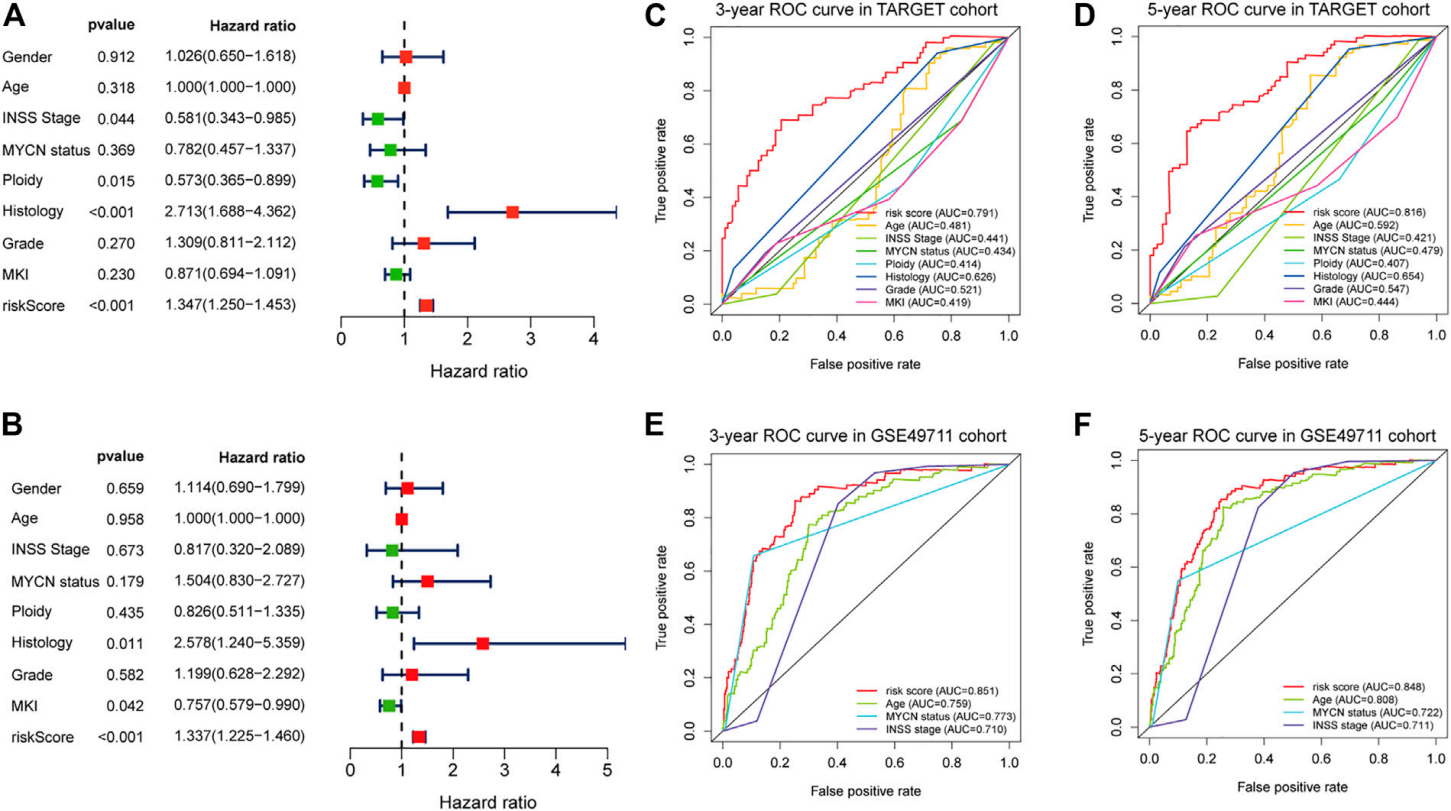
Estimate the predictive ability of the five-RNA-based risk signature. Univariate Cox regression analysis of risk score and clinical factors **(A)**. Multivariate Cox regression analysis of risk score and clinical factors **(B)**. The receiver operating characteristic (ROC) curve for 3-years **(C)** and 5-years survival **(D)** for overall survival (OS) in the TARGET cohort. The ROC curve for 3-years **(E)** and 5-years survival **(F)** for OS in the GSE49710 cohort.

Results of the univariate Cox regression analysis demonstrated that the risk score [HR: 1.370 (95% CI 1.250–1.453), *p* < 0.001], histology [HR: 2.713 (95% CI 1.688–4.362), *p* < 0.001], INSS stage [HR: 0.581 (95% CI 0.343–0.985), *p* = 0.044], and ploidy [HR: 0.573 (95% CI 0.365–0.899), *p* = 0.015] were correlated with OS of NBL patients (Figure 6A). Multivariate Cox regression analysis revealed that risk score [HR: 1.337 (95% CI 1.225–1.460), *p* < 0.001], histology [HR: 2.578 (95% CI 1.240–5.359), *p* = 0.011], and MKI [HR: 0.757 (95% CI 0.579–0.990), *p* = 0.042] were associated with NBL patient survival (Figure 6B). Collectively, the risk score and histology served as independent prognostic factors for NBL. Subsequently, we used the ROC curve and C-index to evaluate the accuracy of our model for predicting the survival of NBL patients. The C-index in the TARGET and GSE49711 cohorts were 0.749 and 0.809, respectively. The ROC analysis showed that our signature performed well in predicting the OS of NBL patients (3-years AUC = 0.791, 5-years AUC = 0.816) in the TARGET cohort (Figures 6C,D). The predictive capability was further validated by the GSE49711 cohort (3-years AUC = 0.851, 5-years AUC = 0.848) (Figures 6E,F).

### Gene Set Enrichment Analysis

**FIGURE 7:**
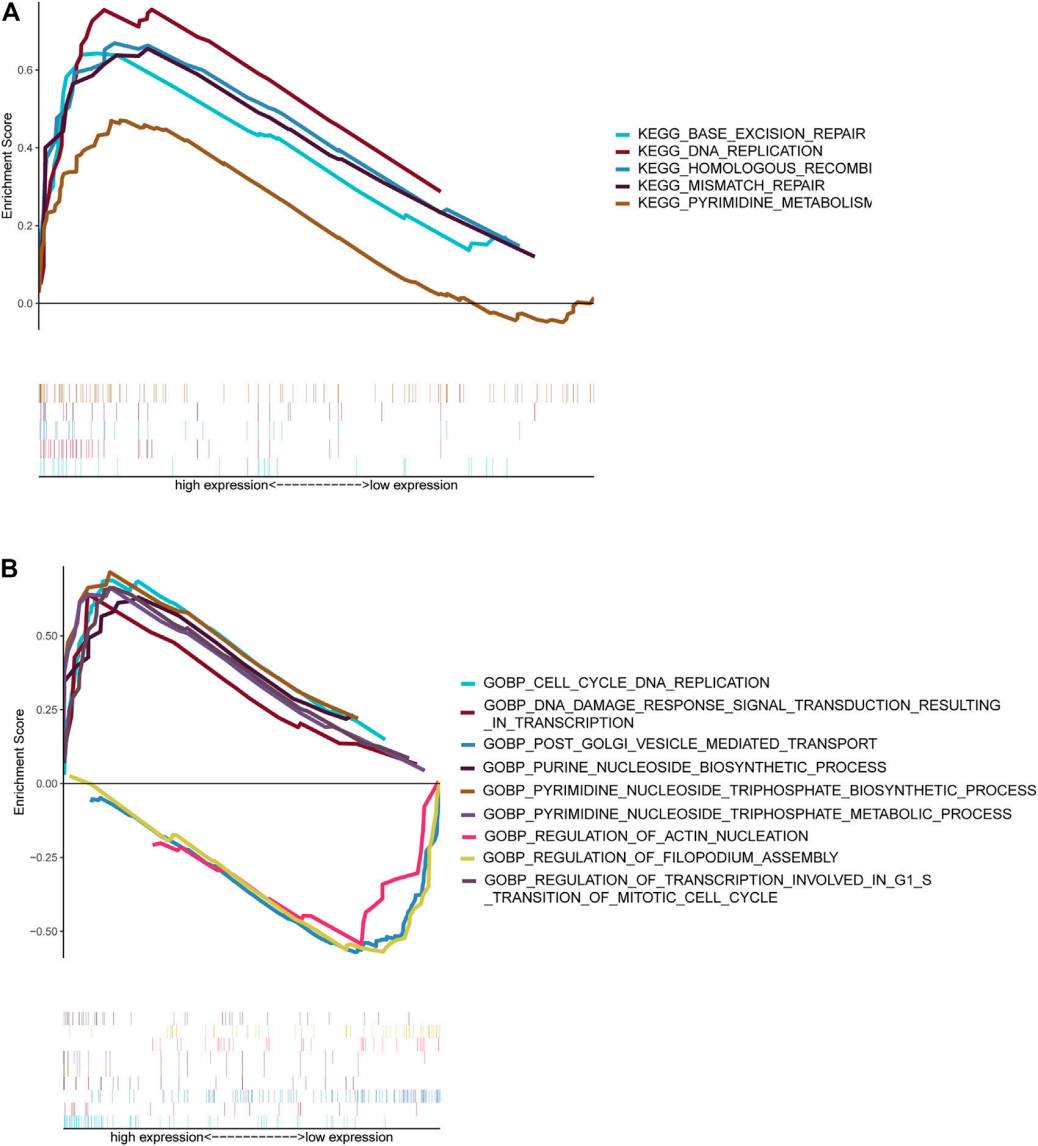
Gene set enrichment analysis. Gene sets enriched in the high-risk score group **(A, C)**. Gene sets enriched in the low-risk score group **(B, D)**. Nom *p*-value < 0.05 was deemed significant.

NBL patients were categorized into two groups based on the median cut-off value of the risk score in the TARGET cohort. GSEA analysis was subsequently performed to investigate the potential downstream signaling pathways relevant to the five RNAs. Gene sets with |NES| >1, NOM *p*-value < 0.05, and FDR <0.25 were displayed. For “c2.cp.kegg.v7.2.symbols.gmt” collection terms, including base excision repair, DNA replication, homologous recombination, mismatch repair, and pyrimidine metabolism, were enriched in the high-risk group (Figure 7A). However, no gene sets were enriched in the low-risk group.

The collection of “c5.go.bp.v7.2.symbols.gmt” (Figure 7B) genes in the high-risk group was primarily enriched in cell cycle–related and pyrimidine nucleoside triphosphate–related pathways. On the contrary, genes were primarily enriched in mitochondrial fission, vesicle-mediated transport, and filopodium assembly in the low-risk group.

### Screening for Candidate Drugs

**FIGURE 8:**
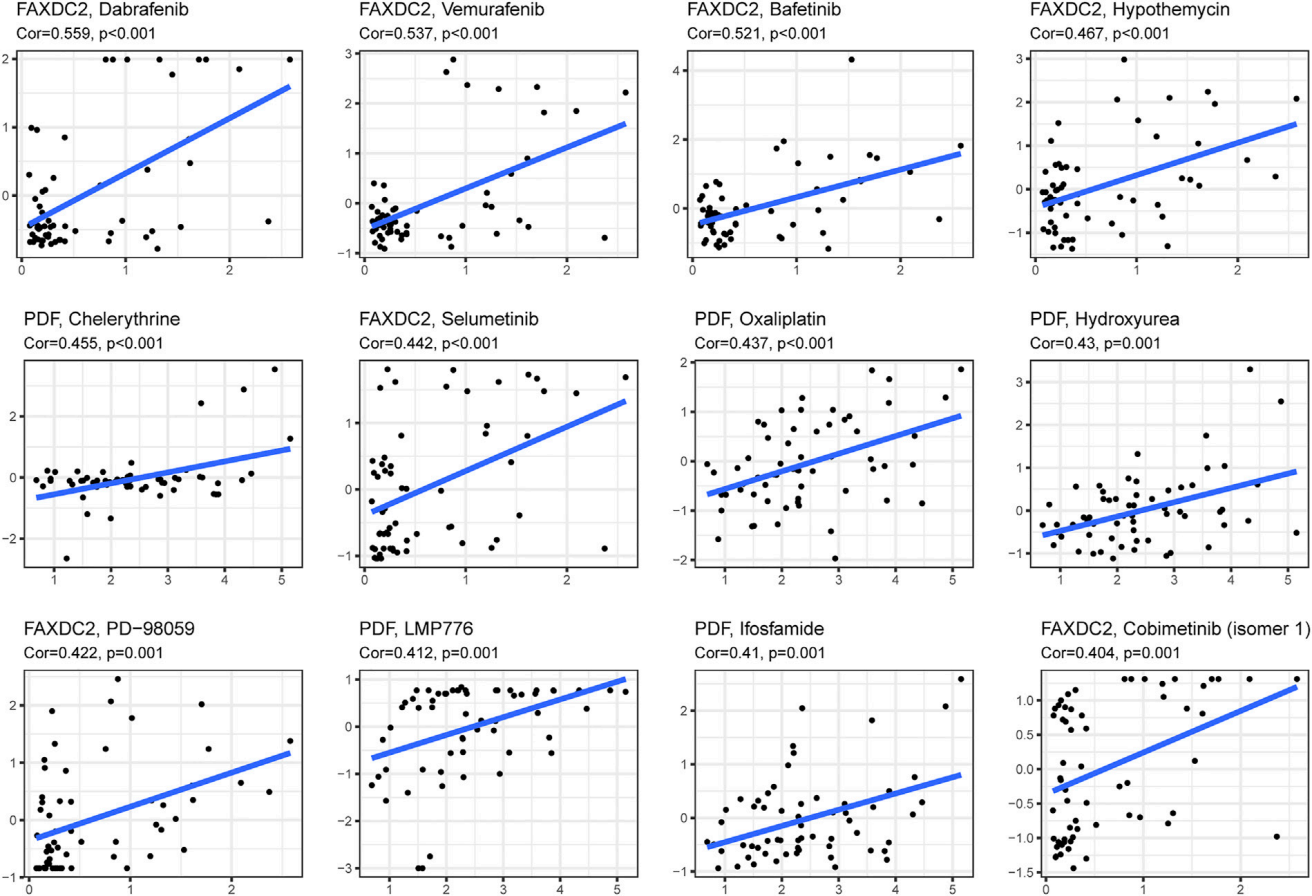
Candidate drugs targeting the five RNAs. The top 12 drugs targeting the five genes ranked by correlation tested by Pearson correlation analysis.

Drug sensitivity and gene expression data of the cell lines were obtained from the CellMiner database. Pearson correlation analysis showed that dabrafenib (R = 0.559, *p* < 0.001), ibrutinib (R = 0.537, *p* < 0.001), and dasatinib (R = 0.512, *p* < 0.001) were correlated with FAXDC2 (Figure 8). In addition, chelerythrine (R = 0.455, *p* < 0.001) was linked to PDF.

## Discussion

NBL is the most common and rare solid tumor with clinical heterogeneity among children. In the United States, approximately 700 children are diagnosed with NBL per year (Li et al., 2008). Although advances in treatment have improved the survival of NBL patients, the prognosis of high-risk NBL remains low (Cohn et al., 2009). Insight into the biology of NBL initiation and progression may help improve the survival of NBL patients. Previous studies have shown that MYCN amplification, copy number alterations, and rearrangements of oncology genes are putative causes contributing to NBL (Fetahu and Taschner-Mandl, 2021). A large-scale study demonstrated that patients aged >18 months at diagnosis had a low survival rate (Moroz et al., 2011). Diverse classifications, including the International Neuroblastoma Risk Group classification (Cohn et al., 2009), and COG risk classification (London et al., 2005), included age as a significant risk factor. These studies suggest that age is a crucial factor in NBL, and understanding the gene expression profile related to age is needed. In this study, we analyzed DEGs between the two age groups. We constructed and validated a five-RNA–based signature to predict the OS of NBL patients.

The DEGs between the two age groups were analyzed based on the 18 months threshold value. Functional enrichment analysis demonstrated that these DEGs may participate in metabolism-related signaling pathways, including retinol metabolism, cholesterol metabolism, tyrosine metabolism, and glycolysis/gluconeogenesis pathways. Previous studies have shown that metabolism could regulate the progression and development of NBL (Fultang et al., 2019; Song et al., 2020). These enriched metabolism-related pathways might imply that metabolism is partially responsible for the heterogeneous outcomes of NBL. Signatures composed of mRNAs, lncRNAs, and miRNAs have been widely developed as valuable tools for predicting cancer prognosis (Chibon, 2013; Kwa et al., 2017). The expression of miRNAs in NBL was extensively downregulated, and 27 miRNAs divided patients into high-and low-risk groups (Lin et al., 2010). An MYCN signature integrated with MYCN activity and chromosomal aberrations exhibited more effective prediction power than MYCN amplification status (Fultang et al., 2019; Song et al., 2020), indicating the suitability and superiority of the risk model construction based on the transcriptome. Using the univariate Cox regression analysis, LASSO regression analysis, and multivariate Cox regression analysis, we selected five RNAs to construct the RNA-based signature for predicting the survival of NBL. Wang et al. identified five genes derived from m6A regulators (METT14, WTAP, HNRNPC, YTHDF1, and IGF2BP2) to construct a risk prediction model that had predictive accuracy (Wang et al., 2020). The effectiveness of our model in predicting 5-years OS (AUCs = 0.879) was less than that of the signature composed of five m6A regulators (AUCs = 0.916) in the GSE49711 cohort. In contrast, the 5-years AUC of our signature (AUCs = 0.816) was higher than that of the m6A-based signature (AUC = 0.739) in the TARGET cohort. Immune-related signatures have been explored, and a five immune-related-gene-based signature (RS5_G) predicted outcomes and groupings (Zhong et al., 2021). We noted that the predictive capability of the five age-related signatures (C-index = 0.809) was close to that of RS5_G (C-index = 0.869) in the GSE49711 cohort. Overall, the five-RNA–based signature displayed equivalent efficiency in assessing the OS of NBL as compared to the published risk models.

Using LASSO screening, 10 RNAs were selected, namely TMUB1, CNR1, TMEM160, FAXDC2, SDF2L1, CTU1, PDF, ULBP1, F8A3, and ANKRD24. These genes contribute to the progression of cancer and are associated with patient outcomes. For example, CNR1, which is expressed primarily in the central nervous system, was reported to regulate the activation of the p38 MAPK pathway, which promoted the progression of HPV-positive head and neck squamous cells (Liu et al., 2020). ULBP molecules are vital ligands of the activating receptor NKG2D on the surface of NK cells. Downregulated ULBP molecules help NBL cells evade the control of the host immune system (Raffaghello et al., 2004). FAXDC2 (C5orf4) is downregulated in acute myeloid leukemia and is associated with the development of megakaryocytes (Jin et al., 2016). The small ubiquitin-like protein encoded by the TMUB1 gene retains p53 in the cytoplasm but decreases nuclear localization, promoting p53-dependent mitochondrial apoptosis (Castelli et al., 2020; Della-Fazia et al., 2020). The expression profiles of SDF2L1, PPP1R12A, and PRKG1 were associated with the clinical outcomes of high-grade serous ovarian cancer. SDF2L1 is an independent prognostic indicator in breast cancer, and the reduced level of SDF2L1 is related to poor clinical outcomes (Jiang et al., 2009). Two signatures of RNA-binding proteins enrolled the CTU1 gene as a vital indicator to predict the survival of prostate cancer and bladder urothelial carcinoma (Guo et al., 2020; Hua et al., 2020). However, the effects of these 10 genes in NBL have not been explored. Carcinoma genome-wide methylation screening revealed that methylation of CNR1 was correlated with MYCN amplification, and patients with low mRNA expression levels of CNR1 had a poor prognosis (Decock et al., 2012). In this study, we noted that CNR1 expression was lower in the MYCN-amplified group, and lower levels of CNR1 were significantly associated with unfavorable outcomes concurring with findings from other studies. Furthermore, differences in PDF, TMEM160, FAXDC2, and F8A3 were observed between the MYCN-amplified and non-amplified groups. MYCN amplification was observed in approximately 20–30% of high-risk NBL patients and is one of the oncogenes in NBL (Brodeur et al., 1977; Pugh et al., 2013; Zhu et al., 2013). Whole-genome analysis suggested that MYCN alterations were more frequently diagnosed in younger patients (Brady et al., 2020), which may explain the correlations of these 10 genes with MYCN status.

In this study, GSEA revealed that DNA replication and homologous recombination are potential NBL initiation and progression pathways. A study on Chinese children observed a strong correlation between genetic variants of the FEN1 gene and neuroblastoma risk (Zhuo et al., 2020) Genomic alterations in DNA damage response–related genes are frequently observed in high-risk NBL (Southgate et al., 2020). Genomic alterations correlate with homologous recombination repair and exist in approximately 50% of 237 NBL patients (Takagi et al., 2017). In addition to the pathways associated with these five RNAs, enhanced ribosome biogenesis activity, directly induced by MYC transcription factors, indicated a poor prognosis of NBL (Hald et al., 2019). Ribosomal RACK1 regulates the expression of cell cycle genes independent of mTOR (Romano et al., 2019). Using the CellMinerCDB database dabrafenib, vemurafenib, and bafetinib, which target RAS-MAPK pathways, were related to the expression of FAXDC2. More frequent mutations in RAS-MAPK pathway-related genes could be detected when NBL relapsed (Valencia-Sama et al., 2020). *In vivo* and *in vitro* dabrafenib was used to treat NBL patients with BRAF V600 mutation (Kieran et al., 2019) and SHP2 inhibitors combined with vemurafenib could treat relapsed neuroblastoma (Valencia-Sama et al., 2020). However, the effect of bafetinib on NBL treatment has not been examined. In addition to the RAS-MAPK pathway, vemurafenib hampers DNA damage repair in melanoma cells (Kimeswenger et al., 2019). Bioinformatic analysis revealed that dabrafenib partially regulates the MUC family’s function, which participates in cell cycle and DNA damage pathways (Jiang et al., 2019). Pathways targeted by candidate drugs are consistent with the signaling pathways identified by GSEA analysis. This study provides possibilities for novel agents for the treatment of NBL.

However, there were some limitations of the present study. First, this study was based on bioinformatic analysis. Thus, a series of *in vivo* and *in vitro* experiments are needed to reveal the biological function of the genes. Second, bias may have existed due to sample selection and grouping. For example, stage 1 and stage 2 NBL patients in the TARGET cohort were excluded because of lack of age records. Thus, variation in the numbers of participants in the two age groups may affect the accuracy of the statistical analysis. Additionally, the efficacy of gene signatures identified from pan-NB populations needs further assessment in different MYCN status subgroups because the MYCN status might confound prognostic signatures (Hallett et al., 2016). Therefore, large-scale and multicenter studies are required to confirm the performance of our study.

In conclusion, we analyzed DEGs between two age groups and constructed a well-performed five-RNA-based signature. Furthermore, we screened candidate drugs targeting five RNAs.

## Data Availability

Publicly available datasets were analyzed in this study. This data can be found here: The TARGET database (www.ocg.cancer.gov/programs/target), The GEO database (www.ncbi.nlm.nih.gov/geo/) and The CellMinerCDB database (www.discover.nci.nih.gov/cellminer/home.do).
