# Supplementary material for: Development and Validation of a Five-RNA–Based Signature and Identification of Candidate Drugs for Neuroblastoma
Source: Front Genet. 2021 Oct 20;12:685646. doi: 10.3389/fgene.2021.685646 (PMC8564070; doi:10.3389/fgene.2021.685646)
Supplement: Supplementary file 1 [file DataSheet1.docx]

| Supplementary Table 1.the correlation of ten RNAs with COG Risk Group（$\bar{X}$±SD） | | | | |
| --- | --- | --- | --- | --- |
| name | High Risk（n=127） | Intermediate Risk（n=13） | Low Risk（n=13） | p |
| ANKRD24 | 1.312±2.154 | 0.403±0.203 | 0.696±0.543 | 0.001 |
| CNR1 | 3.688±3.631 | 10.678±7.604 | 14.034±6.722 | <0.001 |
| CTU1 | 4.347±2.406 | 2.278±1.111 | 1.854±0.488 | <0.001 |
| F8A3 | 0.398±0.796 | 0.035±0.026 | 0.025±0.017 | <0.001 |
| FAXDC2 | 3.429±3.566 | 6.801±2.811 | 11.735±3.596 | <0.001 |
| PDF | 1.713±1.224 | 0.738±0.312 | 0.493±0.084 | <0.001 |
| SDF2L1 | 15.46±9.735 | 7.67±3.847 | 6.792±3.368 | <0.001 |
| TMEM160 | 29.252±18.202 | 13.018±4.424 | 11.934±3.936 | <0.001 |
| TMUB1 | 26.598±10.187 | 13.232±3.742 | 12.019±2.542 | <0.001 |
| ULBP1 | 0.389±0.561 | 1.036±1.16 | 1.198±0.5 | <0.001 |

| Supplementary Table 2.the correlation of ten RNAs with Ploidy（$\bar{X}$±SD） | | | |
| --- | --- | --- | --- |
| name | Diploid (DI=1)（n=66） | Hyperdiploid (DI>1)（n=86） | p |
| ANKRD24 | 1.465±2.366 | 0.962±1.639 | 0.001 |
| CNR1 | 3.545±3.406 | 6.378±6.448 | 0.004 |
| CTU1 | 4.485±2.731 | 3.58±2.005 | 0.015 |
| F8A3 | 0.376±0.625 | 0.308±0.82 | 0.465 |
| FAXDC2 | 3.853±3.509 | 4.796±4.709 | 0.35 |
| PDF | 1.763±1.454 | 1.353±0.923 | 0.032 |
| SDF2L1 | 14.019±8.125 | 14.087±10.532 | 0.488 |
| TMEM160 | 30.19±19.044 | 23.66±16.406 | 0.01 |
| TMUB1 | 24.814±9.722 | 23.889±11.511 | 0.306 |
| ULBP1 | 0.334±0.402 | 0.655±0.811 | 0.07 |

| Supplementary Table 3.the correlation of ten RNAs with INSS Stage（$\bar{X}$±SD） | | | | |
| --- | --- | --- | --- | --- |
| name | Stage 3（n=6） | Stage 4（n=126） | Stage 4s（n=21） | p |
| ANKRD24 | 0.506±0.36 | 1.307±2.165 | 0.626±0.451 | 0.036 |
| CNR1 | 7.799±10.848 | 3.796±3.699 | 12.598±6.539 | <0.001 |
| CTU1 | 2.921±1.658 | 4.323±2.419 | 2.078±0.947 | <0.001 |
| F8A3 | 0.053±0.033 | 0.401±0.798 | 0.024±0.019 | <0.001 |
| FAXDC2 | 4.362±3.323 | 3.409±3.494 | 10.511±3.585 | <0.001 |
| PDF | 0.92±0.329 | 1.718±1.228 | 0.551±0.2 | <0.001 |
| SDF2L1 | 8.766±3.642 | 15.56±9.717 | 6.586±3.358 | <0.001 |
| TMEM160 | 16.746±10.435 | 29.147±18.313 | 12.681±4.002 | <0.001 |
| TMUB1 | 15.95±4.122 | 26.724±10.107 | 11.585±2.603 | <0.001 |
| ULBP1 | 2.302±1.211 | 0.36±0.497 | 0.915±0.578 | <0.001 |

| Supplementary Table 4.the correlation of ten RNAs with MKI（$\bar{X}$±SD） | | | | |
| --- | --- | --- | --- | --- |
| name | High（n=33） | Intermediate（n=41） | Low（n=48） | p |
| ANKRD24 | 1.212±2.097 | 1.029±1.447 | 1.404±2.695 | 0.713 |
| CNR1 | 2.904±3.21 | 4.846±5.514 | 7.114±5.689 | <0.001 |
| CTU1 | 4.019±1.454 | 4.126±2.289 | 3.742±2.568 | 0.138 |
| F8A3 | 0.175±0.489 | 0.408±0.701 | 0.344±0.969 | 0.197 |
| FAXDC2 | 2.643±2.833 | 3.128±3.201 | 6.891±5.023 | <0.001 |
| PDF | 1.462±0.912 | 1.703±1.248 | 1.281±1.078 | 0.056 |
| SDF2L1 | 14.352±10.049 | 13.884±7.675 | 11.913±6.974 | 0.363 |
| TMEM160 | 24.135±11.09 | 29.674±21.682 | 24.084±18.126 | 0.359 |
| TMUB1 | 23.526±7.162 | 27.138±10.956 | 22.093±11.029 | 0.062 |
| ULBP1 | 0.57±0.705 | 0.453±0.7 | 0.644±0.752 | 0.057 |

| Supplementary Table 5.the correlation of ten RNAs with MYCN status（$\bar{X}$±SD） | | | |
| --- | --- | --- | --- |
| name | Amplified（n=33） | Not Amplified（n=119） | p |
| ANKRD24 | 1.153±1.461 | 1.188±2.126 | 0.064 |
| CNR1 | 2.685±2.576 | 5.831±5.909 | 0.001 |
| CTU1 | 4.732±2.355 | 3.762±2.356 | 0.005 |
| F8A3 | 0.453±0.7 | 0.305±0.75 | 0.007 |
| FAXDC2 | 2.236±2.583 | 4.983±4.425 | <0.001 |
| PDF | 2.04±1.406 | 1.39±1.097 | 0.002 |
| SDF2L1 | 13.979±9.093 | 14.079±9.687 | 0.893 |
| TMEM160 | 31.794±19.947 | 25.026±17.006 | 0.029 |
| TMUB1 | 23.075±9.545 | 24.628±11.07 | 0.514 |
| ULBP1 | 0.638±0.616 | 0.482±0.698 | 0.002 |

| Supplementary Table 6.the correlation of ten RNAs with Histology（$\bar{X}$±SD） | | | |
| --- | --- | --- | --- |
| name | Favorable（n=28） | Unfavorable（n=114） | p |
| ANKRD24 | 0.817±1.449 | 1.302±2.179 | 0.006 |
| CNR1 | 11.829±7.161 | 3.686±3.74 | <0.001 |
| CTU1 | 2.216±1.253 | 4.378±2.377 | <0.001 |
| F8A3 | 0.249±1.152 | 0.35±0.615 | <0.001 |
| FAXDC2 | 8.961±4.405 | 3.287±3.431 | <0.001 |
| PDF | 0.72±0.547 | 1.704±1.228 | <0.001 |
| SDF2L1 | 8.17±4.855 | 14.951±9.62 | <0.001 |
| TMEM160 | 12.244±4.173 | 29.268±17.921 | <0.001 |
| TMUB1 | 14.061±7.898 | 26.553±9.462 | <0.001 |
| ULBP1 | 1.088±0.864 | 0.379±0.569 | <0.001 |
